# Digitization of Adsorption Isotherms from “The Thermodynamics and Hysteresis of Adsorption”

**DOI:** 10.6028/jres.126.037

**Published:** 2021-12-16

**Authors:** Daniel W. Siderius

**Affiliations:** 1 National Institute of Standards and Technology, Gaithersburg, MD, USA

**Keywords:** adsorption, hysteresis, isotherm, scanning isotherm

**Data DOI:** https://doi.org/10.18434/mds2-2466

## 1 Summary

Sorption isotherms collected from tables in the seminal dissertation, "The Thermodynamics and Hysteresis of Adsorption" by A. J. Brown [1], have been digitized and made publicly available, along with supporting software scripts that facilitates usage of the data. The isotherms include laboratory measurements of xenon, krypton, and carbon dioxide adsorption (and, when possible, desorption) isotherms on a single sample of Vycor glass^1^1Certain commercially available items are identified in this paper. This identification does not imply recommendation by the National Institute of Standards and Technology, nor does it imply that it is the best available for the purposes described, at various temperatures including subcritical conditions for xenon and krypton. The highlight of this dataset is the collection of "scanning" isotherms for xenon on Vycor at 131 K. The scanning isotherms examine numerous trajectories through the adsorption-desorption hysteresis region, such as primary adsorption and desorption scanning isotherms that terminate at the hysteresis boundary, secondary scanning isotherms made by selective reversals that return to the boundary, and closed scanning loops. This dataset was originally used to test the independent domain theory of adsorption and continues to support successor theories of adsorption/desorption scanning hysteresis including more recent theories based on percolation models. Through digital preservation and release of the tables from Brown's dissertation, these data are now more easily accessible and can continue to find use in developing models of adsorption for fundamental and practical applications.

## 2 Data Specification

**Table:** 

**NIST Operating Unit**	Material Measurement Laboratory, Chemical Sciences Division, Chemical Informatics Research Group
**Format**	CSV (Python supporting software available on Github)
**Data Dictionary**	https://github.com/nist-isodb/AJBrownThesisIsotherms/raw/main/tableschema.json
**Accessibility**	All datasets submitted to *Journal of Research of NIST* are publicly available.
**License**	https://www.nist.gov/director/licensing

Isotherms from Ref. [1] have been digitized in four comma-separated-value (CSV) files available at https://doi.org/10.18434/mds2-2466: three files contain the boundary isotherms of xenon, krypton, and carbon dioxide, in Vycor glass at various temperatures, and one file contains the scanning isotherms of xenon in Vycor at 151 K. (The scanning isotherm file also includes the 151 K boundary isotherm, for convenience.) The filename clearly denotes both the adsorbate fluid and type of isotherm, e.g. xe_boundary_isotherms.csv contains the boundary isotherms of xenon. The data sets are further subdivided into runs. For the boundary isotherms, each run corresponds to a different temperature. For the scanning isotherms, each run corresponds to a continuous sequence of states visited by the particular run; the scanning runs are further subdivided into segments that correspond to different pressure reversal objectives. Metadata about each measurement is included in the row of the data file containing that measurement (i.e., there is no need to cross-reference a row with metadata located elsewhere). The runs and segments are identified and described in metadata columns, so that the purpose of each run and the objective of the pressure sweep is described in situ. The list of data columns and brief description of each column is given in Table 1; the data dictionary is also available online in json-schema format as noted in the Data Specification table.

It is necessary to note that the pressure of measurements in the data files are given in three different forms (as in the original dissertation): "relative" pressure *p/p*_0_, where the absolute pressure measurement has been divided by the saturation pressure of the fluid at the measurement temperature; "log_10_(p/mmHg)", the base-10 logarithm of the measured pressure in mmHg; and unaltered mmHg.

By extracting a set of rows from the file for a particular run (and segment), one can obtain a particular boundary or scanning isotherm and then further process the data as desired for the intended application. Since the files are formatted as CSV files, the data can be accessed using any conventional spreadsheet software. The boundary isotherms have additionally been deposited in the NIST/ARPA-E Database of Novel and Emerging Adsorbent Materials [2], accessible directly at https://adsorption.nist.gov/isodb/index.php?DOI=10.6028/jres.126.037#biblio.

## 3 Methods

The purpose of the dissertation "The Thermodynamics and Hysteresis of Adsorption" by A. J. Brown [1], published in 1963, was to provide careful measurements of adsorption and desorption of various gases in Vycor glass that could test the then-contemporary independent domain theory of hysteresis in adsorption and desorption [3]. Additionally, the laboratory measurements presented in this dissertation uniquely include a set of "scanning" isotherms of xenon in Vycor, covering situations including scanning from the adsorption isotherm, scanning from the desorption isotherm, and scanning paths that include closed

**Table 1:** Description of data columns in the CSV files containing digitized isotherms from Ref. [1].

**Column Name**	**Contents**	**Data Type**
run_id	Identification number of run	Integer
run description	Text description of run	String
segment_id^a^	Identification number of segment of run	Integer
segment_description^a^	Text description of segment	String
adsorbent	Name of adsorbent material	String
adsorbent_mass	Adsorbent mass	Float
adsorbent_mass_units	Units of adsorbent mass	String
adsorbate	Name of adsorbate	String
adsorbate_mass	Molecular weight of adsorbate	String
adsorbate_mass_units	Units of molecular weight of adsorbate	String
temperature_units	Units of temperature measurement (K = Kelvin, C = Celsius)	String
pressure_units	Units of pressure measurement (log10(mmHg), mmHg, or relative)	String
adsorption_units	Units of adsorption measurement	String
temperature	Temperature measurement	Float
pressure	Pressure measurement	Float
adsorption	Adsorption measurement	Float
uncertainty	Uncertainty in final digit of adsorption measurement	Float
branch	Branch of isotherm (A=Adsorption, D=Desorption)	String
notes	Comments, either from original data or editorial notes"	String

a Runs are subdivided into segments only for scanning isotherms.

loops in the hysteresis loop of the boundary isotherm. According to persons familiar with these measurements, these measurements were done painstakingly slowly to ensure that the resultant isotherms (especially the scanning isotherms) were not artifacts of slow adsorption/desorption kinetics or rushed experiments [4]. Unfortunately, the isotherm data in Brown's dissertation were never published in articles dedicated to discussion of the measurements. Certain portions of the data have been included in later publications including a still-influential review by Everett [5], but the result is that valuable measurements of sorption scanning hysteresis were only available in the original dissertation document.

Uniquely, all of the isotherms were presented in the dissertation's appendices in table form, allowing for faithful extraction of the data to a modern digital format. In 2020, Prof. Peter Monson, representing the International Adsorption Society [6], supplied NIST with photocopies of the original data tables from Brown's dissertation and proposed that NIST preserve the data in digital form and make it widely available to the scientific community. These particular photocopies were from the personal scientific notes of Prof. Geoffrey Mason (whose research also addressed adsorption-desorption hysteresis [7-9]) and included helpful hand-written notes about the data that have been preserved in the digitized tables described here. This section presents the conversion of those isotherm data tables to digital files and describes how to access and use the data, ultimately to facilitate its usage for theories of adsorption-desorption hysteresis and scanning hysteresis.

## 3.1 Historical Context of the Isotherm Data

**Fig. 1:**
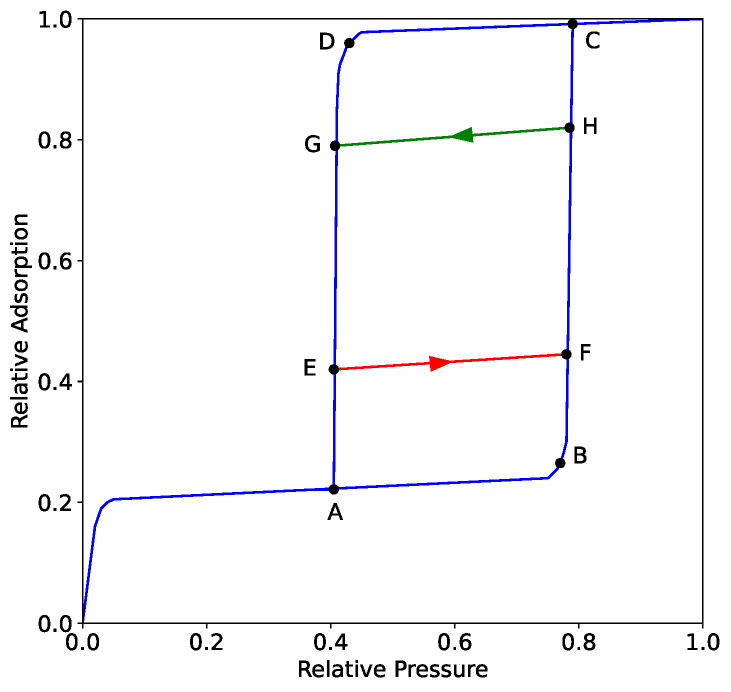
Conceptual schematic of a conventional subcritical adsorption-desorption isotherm with scanning isotherms. The blue traces follow the adsorption and desorption branches of the boundary isotherm. The red trace is a hypothetical adsorption scanning isotherm and the green trace is a hypothetical desorption scanning isotherm. The labeled points identify particular locations in the isotherm to facilitate description in the main test.

The isotherms presented in Brown's dissertation are either *boundary isotherms*, measured with either monotonically increasing or decreasing pressure beginning at either zero pressure or a high pressure where the adsorbent is saturated, or *scanning isotherms*, where the pressure change may purposefully change directions. For example, Figure 1 contains a conceptual adsorption-desorption isotherm (type IV(a), with H1 hysteresis, in the IUPAC classification scheme [10]) at a subcritical temperature of the adsorbate. The blue traces identify the boundary isotherm, which includes a hysteresis loop enclosed by points A, B, C, and D. A scanning isotherm is obtained when an isotherm measurement includes a pressure reversal wherein the subsequent sequence of adsorption or desorption points does not follow the boundary isotherm. In Figure 1, a desorption scanning isotherm is shown by the green trace; the scanning isotherm would be obtained by measuring an adsorption isotherm through points A and B up to point H, at which point the pressure is decreased, stepping through states along the green trace to point G. Similarly, the adsorption scanning isotherm shown in the red trace could be obtained by measuring a desorption isotherm through points C and D to point E, then increasing the pressure to follow a path of states to point F. Other scanning isotherms can follow loops inside the hysteresis loops simply by reversing the pressure at points on and/or inside the hysteresis loop. Scanning isotherms can be used to determine information about pore geometry, network connectivity, and pore-size distribution that is not available exclusively from the boundary isotherm [9]. For a recent work discussing scanning isotherms, consult Ref. [11].

At the time of publication of Brown's dissertation (1963), scanning hysteresis was described via the independent domain theory [3, 5], and the data in the dissertation were used to extensively test the theory. Independent domain theory of adsorption hysteresis may be summarized as follows: a porous material may be composed of an assembly of microscopic domains that behave independently during adsorption and desorption. Each domain can exist in one of two states (filled or empty), and has unique condensation and evaporation pressures where the state changes (e.g., each domain has a unique hysteresis isotherm). An assembly of these microscopic domains will, at some pressure, contain a distribution of some filled domains and some empty domains, which is termed the *domain complexion*. The path traced by the assembly of domains in an adsorption or desorption isotherm is thus determined by the particular sequence of complexions visited by the experiment [12]. Reversing the direction of pressure changes while in a complexion that is neither completely filled nor completely empty can cause the assembly of domains to visit a complexion that differs from the preceding complexion because some domains that changed state in the preceding step may not change state in the reversed step. This non-reversibility that follows from independent behavior of the individual domains results in scanning isotherms that are not kinetic artifacts. For a more complete discussion of independent domain theory, the reader is directed to the recent review in Ref. [12]. Subsequent work highlighted the importance of thermodynamic metastability, pore-blocking, and cavitation as causes of hysteresis, ultimately highlighting the incompleteness of the independent domain theory [10]. While the independent domain theory has been superseded [12, 13], scanning isotherms are still useful for porous material characterization and theoretical modeling when coupled with more modern theoretical interpretations. More recently, percolation models for scanning hysteresis have been developed, where pore connectivity, blockage at pore interconnects, and cascades of pore filling and pore emptying result in scanning hysteresis. Data from Brown's dissertation was used in some of the earliest works on percolation theories of scanning hysteresis [7, 8, 14, 15] and is still referenced today, e.g., Ref. [11].

## 3.2 Data Extraction and Organization

As noted earlier, all of the isotherm measurements in Brown's dissertation were given in table form, greatly easing the process of extracting and organizing the measurements in digital files. Optical character recognition was originally attempted to speed the data extraction process, but this proved infeasible as the character recognition errors were too numerous. Hence, the tables were manually digitized into the CSV files described above. For reference, the isotherm measurements are given in Appendix 3 of the dissertation, divided into tables for each run/segment. The heading of each run/segment includes the name of the adsorbate (xenon, krypton, or carbon dioxide), the name of the adsorbent (Vycor, in all cases), and the temperature in kelvins. The data follows in three columns: a character descriptor of the isotherm branch (either A=adsorption or D=desorption), the measured mass of adsorbate in mg with an estimate of the uncertainty in the last digit of the adsorbed mass, and the pressure of the measurement. The other metadata necessary to describe the experiment includes the mass of Vycor (which was 61.60(6) mg in all experiments; all used the same sample of Vycor). The original data tables also include various annotations about equipment issues associated with specific measurements and indications that saturation had been achieved; these annotations are included in the 'notes' column of the CSV files. Thus, it was straightforward to convert the data tables in Appendix 3 to the expanded tables in the digital CSV files, where the fully descriptive metadata and explanatory notes are included with each triplet of (isotherm branch, adsorbate mass, pressure). [augmented data] Lastly, the copy of Appendix 3 supplied to the author of this manuscript contains various hand-written notes, pointing out concerns about the data; these notes and other similar comments by the present author (marked "Editor") have also been included in the 'notes' column of the CSV file.

## 3.3 Data Processing Tools

Given both the large set of data and the multiplicity of modes for presenting pressure measurements, Python scripts have been developed for processing the isotherms in the CSV files. Most importantly, there are scripts that:

1. Provide the saturation pressure (*p*_0_) of xenon and krypton according to the correlations used in Ref. [1];
2. Extract a single isotherm run and segment (if applicable) from the CSV file;
3. Convert the isotherm to a consistent format, where the pressure is given in absolute units and the adsorption has been normalized by the adsorbent mass;
4. Convert the isotherm to the NIST JSON Isotherm format [2];
5. Extract a set of scanning isotherms and plotting the resultant set;
6. Silently preserve the number of significant figures in converted data, particularly for conversion of the logarithmic pressure to nominal pressure;
7. Convert the pressure measurements to other desired units.

## 3.4 Example Data Processing

As examples of how to use the data processing tools, three Jupyter Notebooks are provided with the supporting software:

Example_Processing.ipynb: A brief demonstration of extraction of the boundary isotherm and one primary scanning isotherm of xenon;

Boundary_Isotherms.ipynb: An extensive demonstration of how to extract and manipulate the boundary isotherms;

Scanning_Isotherms.ipynb: A demonstration of how to extract scanning isotherms either individually or in sets and then plot the isotherm(s).

## 4 Impact

The sorption isotherms, both boundary and scanning types, from A. J. Brown's dissertation [1] have been in use for nearly 60 years, supporting the development of successive theories of adsorption-desorption hysteresis and scanning hysteresis over that time. By preserving those isotherm measurements and making them more widely available, the intention is that these data continues to support theoretical models of hysteresis and pore characterization from isotherm measurements.
